# A New Epitope Selection Method: Application to Design a Multi-Valent Epitope Vaccine Targeting HRAS Oncogene in Squamous Cell Carcinoma

**DOI:** 10.3390/vaccines10010063

**Published:** 2021-12-31

**Authors:** Kush Savsani, Gabriel Jabbour, Sivanesan Dakshanamurthy

**Affiliations:** 1 College of Humanities and Sciences, Virginia Commonwealth University, Richmond, VA 23284, USA; savsanikv@mymail.vcu.edu; 2 School of Medicine, Georgetown University Medical Center, Washington, DC 20057, USA; gmj15@georgetown.edu; 3 Molecular and Experimental Therapeutic Research in Oncology Program, Lombardi Comprehensive Cancer Center, Georgetown University Medical Center, Washington, DC 20057, USA

**Keywords:** epitope selection method, epitope-based vaccine, immuno-informatics, MHC molecules, HLA alleles, design of T cell epitope-based vaccine candidate, multivalent epitopes, HRAS epitopes, squamous cell carcinoma vaccine

## Abstract

We developed an epitope selection method for the design of MHC targeting peptide vaccines. The method utilizes predictions for several clinical checkpoint filters, including binding affinity, immunogenicity, antigenicity, half-life, toxicity, IFNγ release, and instability. The accuracy of the prediction tools for these filter variables was confirmed using experimental data obtained from the Immune Epitope Database (IEDB). We also developed a graphical user interface computational tool called ‘PCOptim’ to assess the success of an epitope filtration method. To validate the filtration methods, we used a large data set of experimentally determined, immunogenic SARS-CoV-2 epitopes, which were obtained from a meta-analysis. The validation process proved that placing filters on individual parameters was the most effective method to select top epitopes. For a proof-of-concept, we designed epitope-based vaccine candidates for squamous cell carcinoma, selected from the top mutated epitopes of the HRAS gene. By comparing the filtered epitopes to PCOptim’s output, we assessed the success of the epitope selection method. The top 15 mutations in squamous cell carcinoma resulted in 16 CD8 epitopes which passed the clinical checkpoints filters. Notably, the identified HRAS epitopes are the same as the clinical immunogenic HRAS epitope-based vaccine candidates identified by the previous studies. This indicates further validation of our filtration method. We expect a similar turn-around for the other designed HRAS epitopes as a vaccine candidate for squamous cell carcinoma. Furthermore, we obtained a world population coverage of 89.45% for the top MHC Class I epitopes and 98.55% population coverage in the absence of the IFNγ release clinical checkpoint filter. We also identified some of the predicted human epitopes to be strong binders to murine MHC molecules, which provides insight into studying their immunogenicity in preclinical models. Further investigation in murine models could warrant the application of these epitopes for treatment or prevention of squamous cell carcinoma.

## 1. Introduction

Squamous cell carcinoma (SCC) is a form of non-melanoma skin cancer. It begins in squamous epithelial cells that form the surface of the skin, the surface of organs, and the lining of the respiratory and digestive tract. It is the second most common form of nonmelanocytic skin cancer after basal cell carcinoma, accounting for 20% of cutaneous malignancies and 75% of all non-melanoma skin cancer deaths [1]. UV radiation and other DNA-damaging agents in squamous cells can lead to SCC. The 5-year survival rate of SCC is ≥90%, rendering a favorable prognosis. Traditional therapies such as Mohs surgery can be effective in removing malignant squamous cells to prevent the spread of the cancer. Additional treatment procedures include curettage, electrodessication, cryosurgery, laser surgery, and radiation therapy. Immunotherapeutic drugs are available for metastasized cases of cutaneous SCC, including cemiplimab and pembrolizumab, which both target lymphocytes PD-1 receptors. However, these checkpoint inhibitors produce durable responses in only <15–20% of patients [1].

The HRAS gene is a proto-oncogene and encodes for the H-Ras protein [2] which binds to GTP and GDP [3]. The H-Ras protein undergoes exchange between the cell’s plasma membrane and Golgi apparatus. This exchange is regulated by cyclic de- and re- palmitoylation. The H-Ras is involved in membrane trafficking, cell survival, calcium signaling, and cellular apoptosis. Mutations to the HRAS gene have been associated with several forms of cancer and other diseases such as Costello syndrome. According to the American Association for Cancer Research (AACR), the HRAS proto-oncogene is involved in 3.5% of squamous cell carcinoma cases involving the head and neck, and more generally in 2.5% of all squamous cell carcinoma cases [4]. In addition, HRAS mutations have been reported in 15% of patients during acquisition of resistance to cetuximab [1], an epidermal growth factor receptor inhibitor. Several mutations to the HRAS gene are considered to result in SCC growth. In a study conducted on 51 squamous cell papilloma patients, four main mutations were identified, occurring on the 12th, 13th, and 61st codons (G12D, G13R, G13V, and Q61L) [5]. The most predominant mutation found in patients was the Q61L mutation, present in 82% of HRAS cases. Other mutations to the HRAS also occur, especially common on the 12th and 13th codons.

Previous studies have been conducted on the immune response in the presence of mutated HRAS peptides [6]. Mice which were treated with an epitope-based vaccine containing the G12A HRAS mutation were protected from developing tumors with the G12A mutation, but not from tumors harboring other RAS mutations. Furthermore, mice that had CD8+ T cell depletion did not have protection against developing tumors. In another study, mutant RAS peptides were administered to patients along with granulocyte-macrophage colony-stimulating factor to stimulate a T-cell immune response. Immune responses were observed “in the majority of patients” [7]. However, it has been difficult to obtain anti-tumor responses from such epitope-based vaccines thus far. These epitope-based vaccines were administered to patients in end-stage metastatic SCC, so rates of survival were predictably low.

In this study, we considered several important clinically relevant factors when designing an epitope-based vaccine (immunogenicity, antigenicity, half-life, toxicity, IFNγ release, allergenicity) to increase the vaccine’s chances of success in pre-clinical and clinical trials [8]. An intramuscular epitope-based vaccine that targets HRAS would require such clinical checkpoints to be met. Primarily, the peptides that compose the vaccine would require strong binding affinity for the human leukocyte antigen (HLA) molecules in a large population. The peptide would slowly release into the bloodstream from its injection site and enter the endoplasmic reticulum (ER), where the peptide is spliced and packaged with HLA molecules for extracellular expression [8]. Interaction between the peptides expressed on these HLA molecules and TCR complexes on CD8+ and CD4+ T-cells will induce an initial immunogenic response and a later antigenic response in the presence of the highly-expressed HRAS mutations. Additionally, they would need to cause an immunogenic as well as antigenic response, induce IFNγ secretion, have a long half-life in vivo, and express non-toxicity, non-allergenicity, and stability [9]. Immunogenicity and antigenicity are necessary to ensure that the peptides elicit an adequate immune response in the short and long-term. IFNγ release (either positive or negative release) provides a strong indication of the likelihood and strength of an immune response. Toxicity and allergenicity are necessary parameters to determine viability in vivo, as toxic or allergenic peptides will not be considered for further stages of drug development. Half-life is a strong gauge of potential success in vivo, as a longer half-life will lead to a greater likelihood of an immune response. Furthermore, predicting the murine MHC binding affinity allows for the study of these peptides in preclinical murine models.

We assessed the accuracy of several tools to predict these clinically-relevant variables and optimized the top epitope determination process through a new epitope selection method. We then predicted these clinically relevant variables for the most common mutated epitopes of the HRAS gene. These variables allowed us to determine the most prevalent and relevant epitopes in designing an epitope-based vaccine against SCC. Previously, several studies have been conducted using a combination of most of these variables [8,9,10,11]. However, antigenicity and the instability index in the filtration of cancer-related peptides were not considered. Our study is the first to combine all clinically relevant parameters to design an epitope selection method, as well as consider the potential success in murine preclinical trials.

## 2. Materials and Methods

### 2.1. Obtaining Mutated HRAS Peptide Sequences

We obtained the unmutated HRAS peptide sequence from the National Library of Medicine [12]. We then produced 44 mutated sequences of the HRAS gene using the top 15 HRAS mutations (G12C, G12D, G12S, G12V, G13C, G13D, G13R, G13S, G13V, A59T, Q61H, Q61L, Q61R, and E62G). Some of these mutated sequences included multiple mutations on adjacent codons (e.g., G12C + G13D).

### 2.2. MHC Class I Binding Affinity Determination

Epitopes for each of the mutations were obtained through IEDB NetMHCpan EL 4.1 [13]. NetMHCpan EL 4.1 returns epitopes along with their predicted binding affinity using two metrics for the top 27 expressed HLA alleles in the human population [14,15]. The first metric is a score between zero and one based on the IC50 value. The second metric is a percentile rank of the predicted eluted ligand likelihood score compared to a set of random natural peptides. We used the percentile rank methodology reported by Dhanda, et al., as an indicator of binding affinity rather than score [16].

Next, we computed several epitope specific clinical checkpoint parameters listed in Table 1. Immunogenicity was determined using the IEDB Class I Immunogenicity Tool [17]. This tool determines the immunogenicity of a peptide based on non-anchor position amino acids, which are the amino acids that play a less significant role in the binding affinity of the peptide. Each non-anchor amino acid is given a log enrichment score, which is related to the ratio of the fraction of that amino acid in an immunogenic dataset to a non-immunogenic dataset. The immunogenicity score is the sum of these log enrichment scores for all non-anchor amino acids.

Antigenicity was determined using VaxiJen v2.0 [18]. VaxiJen antigenicity predictions are based on autocross covariance transformations of protein sequences into uniform vectors of principal amino acid properties. When tested against a dataset of tumor antigens, VaxiJen returned more than 85% accuracy.

Toxicity was determined with ToxinPred [19]. It is based on the finding that certain amino acid residues are more prevalent at certain positions on toxic peptides. ToxinPred uses a dipeptide model based on machine learning and quantitative matrices to predict peptide toxicity with an accuracy of 94.50%.

IFNγ release was predicted with IFNepitope [20]. IFNepitope produces predictions based on peptide length, positional conservation of residues, and amino acid composition. Using a hybrid approach of both machine learning and motif-based prediction, IFNepitope can predict IFNγ release to 82.10% accuracy.

Allergenicity scores were collected using the AllerTop v2.0 tool [21]. The inability to run batch data on the AllerTop v2.0 tool is one shortcoming that prevents the rapid collection of allergenicity data. To solve this issue, we developed a web scraper in Python to automatically pull data from the AllerTop website given an input of epitope data (Supplementary Py Program 1). The allergenicity data is printed in a copiable format, which can be pasted directly in Excel.

Half-life, isoelectric point, instability index, aliphatic index, and GRAVY score were all predicted with ProtParam [22]. We also developed a tool to pull all epitope data from the ProtParam website (Supplementary Py Program 2). All data for unfiltered epitopes can be found in Supplementary Table S1.

### 2.3. Selectin of Top Epitopes

**Figure 1:**
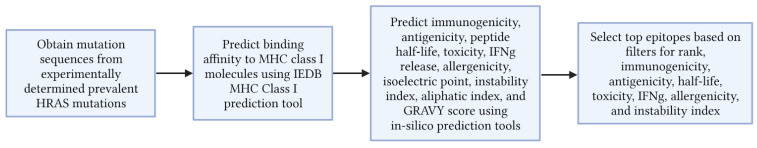
Overall flow of procedures to collect and filter epitope data. Once mutation data is obtained, MHC class I epitope data can be collected from various listed sources. Epitopes can then be filtered to select for clinically viable top epitopes.

The finalized thresholds for the epitope selection process were decided based on previous studies [6,7]. Thresholds were selected to provide sensitivity high enough to obtain a large number of high-quality epitopes while not sacrificing sensitivity. Individual filters were placed on each variable following the findings of the different tests using a dataset of experimentally validated epitopes. The dataset comprised SARS-CoV-2 data, since the dataset provided a vast number of immunogenic and non-immunogenic epitopes based on a metanalysis of several studies using MHC class I epitopes. Top epitopes were then determined based on the combination of filters (Table 1). Figure 1 outlines the flow of procedures to collect peptide data and select the top epitopes.

### 2.4. Murine Model Binding Affinity and Three-Dimensional Analysis

The binding affinity of top epitopes for murine MHC molecules was predicted to determine immunogenicity of the vaccine in pre-clinical murine models. Murine MHC binding affinity was predicted using NetH2pan [23]. Additionally, world and regional population coverage were predicted for the top epitopes using IEDB Population Analysis tool [24]. We also predicted the three-dimensional structure (3D) of the MHC HLA allele-peptide-TCR complex. The three-dimensional structural analysis of the peptide and its MHC complex reveal how the peptide binds to the binding site of the MHC molecule. This is further useful in the three-dimensional analysis of the peptide binding affinity. HLA allele structure was obtained from the RCSB protein data bank [25] and the peptide 3D structure was predicted from PEPstrMOD [26]. Spatial docking of the peptide in the MHC groove was conducted using PyMOL and FlexPepDoc [27].

## 3. Results

### 3.1. Immune Epitope Database (IEDB) Binding Affinity Analysis

Epitopes for the 44 mutated sequences of the HRAS gene were obtained using IEDB NetMHCpan EL 4.1. Epitopes of length 9 and 10 were obtained from the mutated sequences, and binding affinity was determined to the top 27 HLA alleles present in the human population. These alleles are: HLA-A*01:01, HLA-A*02:01, HLA-A*02:03, HLA-A*02:06, HLA-A*03:01, HLA-A*11:01, HLA-A*23:01, HLA-A*24:02, HLA-A*26:01, HLA-A*30:01, HLA-A*30:02, HLA-A*31:01, HLA-A*32:01, HLA-A*33:01, HLA-A*68:01, HLA-A*68:02, HLA-B*07:02, HLA-B*08:01, HLA-B*15:01, HLA-B*35:01, HLA-B*40:01, HLA-B*44:02, HLA-B*44:03, HLA-B*51:01, HLA-B*53:01, HLA-B*57:01, and HLA-B*58:01. The binding affinity data obtained from IEDB is available in Supplementary Table S1. 

### 3.2. Determining Superior Allergenicity Predictor

**Figure 2:**
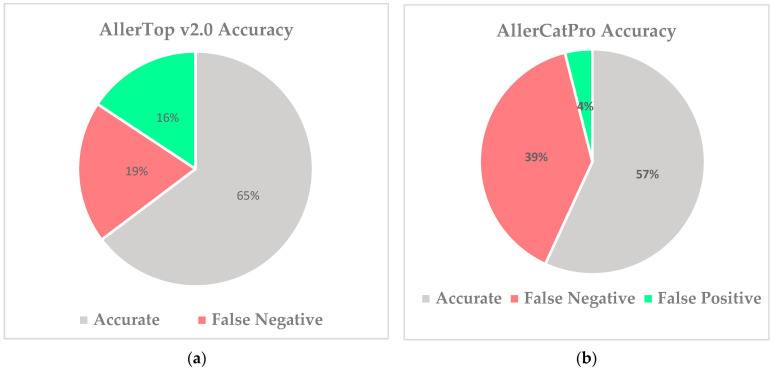
Comparison of allergenicity prediction tools using experimental allergenicity data from IEDB. (**a**) AllerTop v2.0 demonstrated superior sensitivity with a higher accuracy and higher false positive rate. (**b**) AllerCatPro demonstrated a greater rate of false negatives. AllerTop v2.0 was deemed the optimal allergenicity prediction tool for MHC class I epitope data prediction.

Allergenicity scores were computed using the AllerTop v2.0 tool, which was compared directly against AllerCatPro [28] using experimental data. The data were retrieved from the IEDB, resulting in a total of 51 epitopes that were characterized as either allergenic or non-allergenic. Allergens predicted to be non-allergenic by either tool, AllerTop v2.0 or AllerCatPro, were considered false negatives. Non-allergens predicted to be allergens by either tool were marked as false positives. AllerTop v2.0 demonstrated 65% accuracy rate with 16% false positive rate and 19% false negative rate, respectively. AllerCatPro demonstrated 57% accuracy rate with 4% false positive rate and 39% false negative rate (Figure 2).

### 3.3. Replacement of “Cell Permeability” Parameter

We initially considered cell permeability as one of the parameters in the process of determining top epitopes. Cell permeability is an important factor to consider in peptide vaccine design as it directly relates to the vaccine’s ability to be delivered in cells. We initially predicted an epitope’s cell permeability using the tool CellPPD [29]. CellPPD has very low sensitivity for cell penetrating peptides, so a threshold value of −0.5 was used to increase sensitivity. However, this tool returned cell penetrance based on the peptide length rather than considering amino acid composition. Therefore, we eliminated the parameter and replaced it with other related metrics from ProtParam, such as the GRAVY score.

### 3.4. Formula Derivation Using a Two-Variable Equation

To select the top epitopes that are most likely to elicit the desired long-term immune response and maximize efficacy, epitopes were selected based on a series of thresholds and filters. A two variable equation is described by Equation (1):

“Score” = α (“Immunogenicity”) + (1-α) (“Percentile rank”) (1)

In Equation (1), the immunogenicity score and percentile rank are normalized [30]. The α value represents the weight placed on the immunogenicity score when selecting top epitopes (a higher α value means that more weight is placed on the immunogenicity score). An α value of 0.4 was used, although it is stated that an optimal value for α is between 0.4 and 0.6. To determine the optimal α value and the greatest number of high-quality epitopes, α values of 0.4, 0.5, and 0.6 were used for predicted epitopes of the top four HRAS gene mutations.

After applying Equation (1) to all epitopes for the top four HRAS mutations, the top 100 epitopes based on highest score were selected. Before using Equation (1) to select top epitopes, the epitopes were not filtered based on percentile rank. Using this filtration method resulted in epitopes with high, undesirable percentile rank scores with values greater than ten. Starting with a percentile rank filtration step and following it by sorting epitopes based on total formula score will result in epitopes with more favorable rank scores. An α value of 0.4, which places a greater emphasis on percentile rank, resulted in a greater number of unique epitopes. Importantly, the higher number of unique epitopes are not guaranteed and, therefore, it is necessary to repeat the same procedure using a greater abundance of experimental data. Additionally, placing greater emphasis on rank resulted in a greater number of non-immunogenic epitopes (immunogenicity less than 0). Fleri et al. showed that the variables of rank and immunogenicity are independent of each other, so formulaic determination of top epitopes using both variables does not provide “vastly improved predictions” [31]. Fleri’s et al. suggestions provide another method to filter and select top epitopes, which would disregard any formulaic determination and rather utilizes individual thresholds. The epitope selection method that we designed in this study utilizes Fleri’s method by placing individual thresholds on each of our clinical parameters. For this study, we selected top epitopes using the thresholds outlined in Table 1.

### 3.5. Epitope Optimization for Maximum Population Coverage

**Figure 3:**
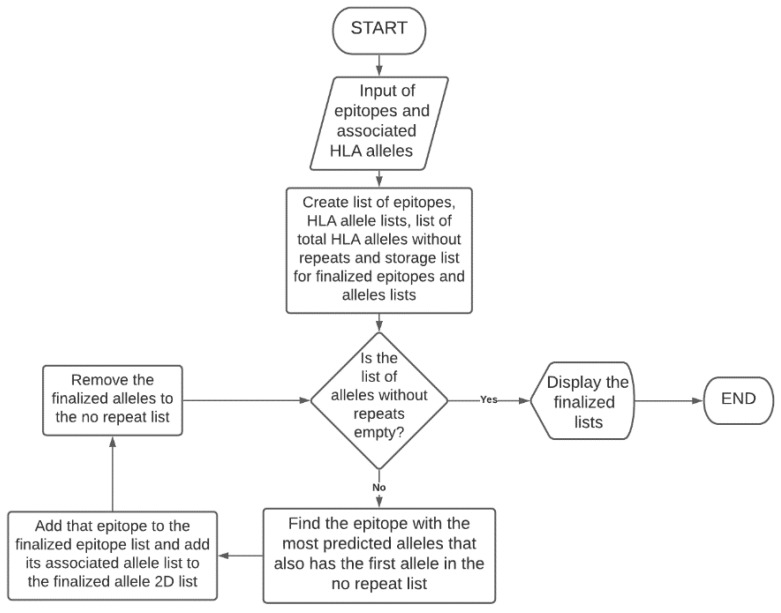
Logical work flow process of PopCoverageOptimization program to optimize peptide selection. The program obtains maximum population coverage by selecting for epitopes that bind to each predicted HLA allele. Small epitope count is sacrificed for population coverage in this model.

We developed a program called ‘PopCoverageOptimization’, that collects all epitopes and their associated HLA alleles as input, and returns the lowest number of epitopes required to obtain maximum possible population coverage as output (Figure 3).

By utilizing this program, we can determine the minimum number of epitopes needed to be returned from the filtration method. This will help in determining the optimal filtration method. Specificity should be prioritized over sensitivity so that the likelihood of selecting successful epitopes is maximized. Hence, if a filtration method results in fewer epitopes than other filtration methods, but still returns the maximum possible population coverage, that method is considered the most successful.

**Figure 4:**
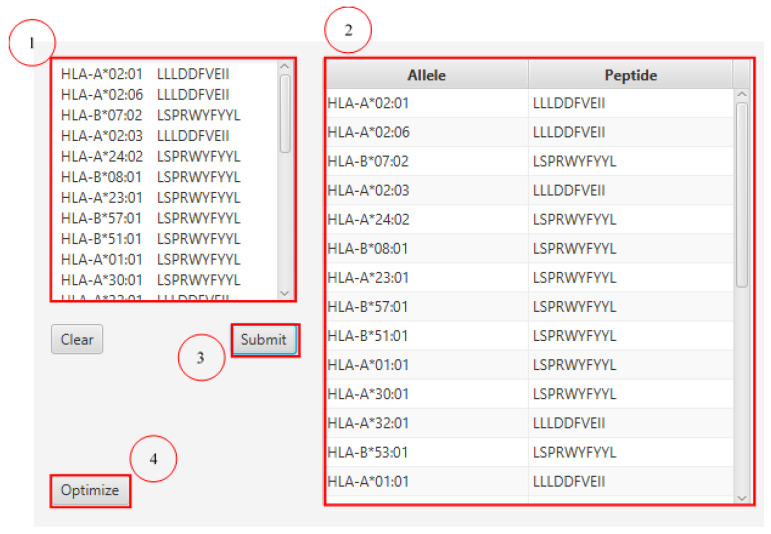
Home screen of PopCoverageOptimization (PCOptim) graphical user interface (GUI) system. Raw epitope data can be inputted in the left-most textbox. After submitting the input, the data will appear formatted in the right-most table. Clicking the “Optimize” button will display the optimized data.

**Figure 5:**
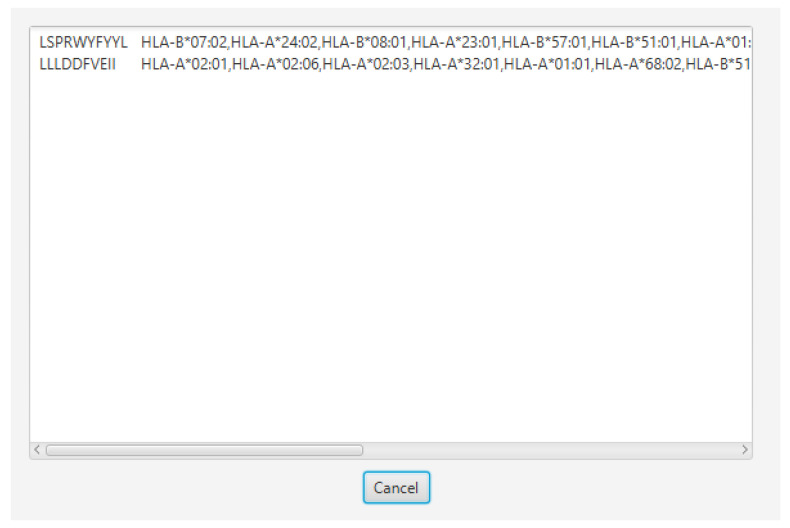
Optimization screen of PCOptim GUI system. Optimized data can be copied and pasted into a.txt file for population coverage prediction or pasted into a spreadsheet to save.

We also designed a graphical user interface using JavaFX for ‘PopCoverageOptimization’, named as ‘PCOptim’, to allow for greater user access to the optimization algorithm. Users can enter their data into the text box labelled 1 in Figure 4. Data from excel should include the predicted HLA allele in the column preceding the peptide sequence column. Clicking the “Optimize” button will reveal a screen with the optimized epitopes returning the maximum possible population coverage given the input data (Figure 5). The PCOptim GUI and source code can be found in Supplementary Java Prog 1. More automation java programs can be found in Supplementary Java Prog 2.

### 3.6. Consideration of a Three-Variable Filtration Method

Adding to the previous methods of determination of top epitopes (two-variable equation and individual thresholds), resulted in a three-variable equation (Equation (2)).

“Score” = x (“percentile rank”) + y (“immunogenicity”) + z (“antigenicity”)(2)

where x + y + z = 1. Three variations of the equation were tested: 0.2/0.4/0.4, 0.3/0.35/0.35, and 0.33/0.33/0.33 as the ratios for x/y/z respectively. For all three formula scenarios, the percentile rank was initially filtered to return only epitopes with a percentile rank of less than or equal to five. This initial filtration step preemptively places emphasis on the percentile rank, which is why less weight is placed on the rank within the formula. Additionally, another method was considered where individual filters were placed on the variables of percentile rank, immunogenicity, and antigenicity which is explained in the next section

### 3.7. Validation of Epitope Selection Method Using Experimental Immunogenic Epitopes

Ultimately, four filtration methods were compared. The first three methods all used a variation of the three variable equation using different values for x, y, and z (0.2/0.4/0.4, 0.3/0.35/0.35, and 0.33/0.33/0.33). The fourth method was an individual filtration step that selects top epitopes using the thresholds outlined in Table 1. The determination of the success of filtration methods did not provide meaningful results when filtration testing was performed with predicted epitopes rather than experimental epitopes. To determine the success of the finalized four filtration methods (three ratios and individual filtration method), experimental data from the Quadeer et al. database was used [32]. The Quadeer et al. meta-analysis was utilized for experimental immunogenic epitopes to design a filtration method that provide top, high-quality epitopes post-filtration. Quadeer et al. conducted a meta-analysis of SARS-CoV-2 T cell epitope experimental data from 18 studies, which provides experimentally determined immunogenic and non-immunogenic T-cell epitopes for SARS-CoV-2. The database provides the option to sort through all data (overall), solely the immunogenic data, and sort by protein. Of the 18 studies providing T-cell epitopes, the immunogenic epitopes of length 9 or 10 came from a study conducted on the Spike protein by Snyder et al. [33]. The study utilizes three acutely infected and 58 recovered patients of SARS-CoV-2, as well as performing T-cell repertoire sequencing on 1815 samples from a total of 1521 subjects. 

Only epitopes with a length of 9 or 10 amino acids long were obtained from the filtration of the overall epitope data. This resulted in a total of 640 epitopes. These epitopes were associated with specific HLA alleles. We then determined the MHC binding affinity of the epitopes using IEDB NetMHCpan EL 4.1. Epitopes with both matching amino acid sequences and MHC alleles were selected, resulting in 97 epitopes. Immunogenicity and antigenicity data were predicted for the remaining epitopes, and percentile rank, immunogenicity, and antigenicity were all normalized. Percentile rank was normalized using Equation (3), while immunogenicity and antigenicity were normalized using Equation (4).

(3) $$\mathrm{rank}\_\mathrm{norm}=\left(-1\times \frac{\mathrm{value}-\min}{\max-\min}\right)+1$$

(4) $$\mathrm{norm}=\frac{\mathrm{value}-\min}{\max-\min}$$

Full data for the immunogenic SAR-CoV-2 epitope study can be found in Supplementary Table S2. The individual filtration step was determined to be the optimal filtration strategy using ‘PopCoverageOptimization’. The 134 epitopes were run through the program, which returned four unique peptide sequences. Since the individual filtration step returned five unique epitopes, we determined that the individual filtration step was adequate in returning a high population coverage.

### 3.8. Finalized Filtration

The overall finding of the procedure using experimental SARS-CoV-2 epitopes was that the optimal filtration method is the one which utilizes individual filters on parameters. The half-life of the epitopes was still not a parameter considered. We solved this issue by using ‘ProtParam’ to find the half-life of the peptides [22]. After collecting the SARS-CoV-2 epitopes, 20,359 epitope-HLA allele pairs were found to match the spike protein genome. After filtering the data using the finalized filtration method, 67 epitope-HLA allele pairs remained.

We selected top epitopes of the 15 HRAS mutations by placing individual thresholds on each clinically relevant parameter as our filtration method (outlined in Table 1). We obtained 16 unique peptide sequences which account for nine unique HRAS mutations after filtering with the aforementioned thresholds. Each epitope is predicted to bind several HLA alleles, which correlates to a high population coverage. The selected top 16 epitopes are provided in Table 2. A complete dataset of top epitopes with the clinically relevant variable predictions is available in Supplementary Table S3.

### 3.9. Population Coverage of Top Epitopes

**Figure 6:**
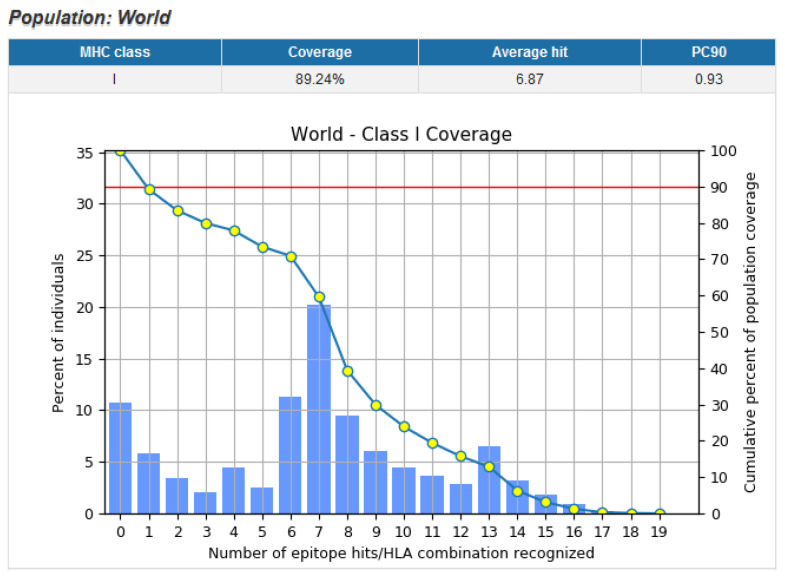
Population coverage graph for world population. The top epitopes demonstrate 89.24% population coverage when filtered for IFNγ release. The pc90 indicates the minimum number of hits required to obtain 90% coverage, which indicates that potential optimization of the top epitope list is possible. Retrieved from IEDB, “tools.iedb.org/population” (accessed on 24 September 2021).

Utilizing this data, and using the IEDB population coverage analysis tool, we determined population coverage of a vaccine that is only composed of the top epitopes (Figure 6). The overall population coverage for the world population was estimated to be 89.24%. The estimated population coverage is calculated based on the prevalence of HLA alleles predicted to bind to the provided epitopes. Data for smaller subsets of the population can also be determined using the IEDB population coverage prediction tool. The regional data illustrates the effectiveness of an epitope-based vaccine in different areas of the world (Table 3). The populations in these different areas have differing genetic make-up contributing to differing HLA allele spread. The estimated population coverage for Europe and North America is adequate (about 90% or more), while the coverage for other regions of the world vary from extremely low (2.78% for Central America) to almost adequate (88.11% for the West Indies).

### 3.10. Top Epitope Selection without IFNγ Release Parameter

**Figure 7:**
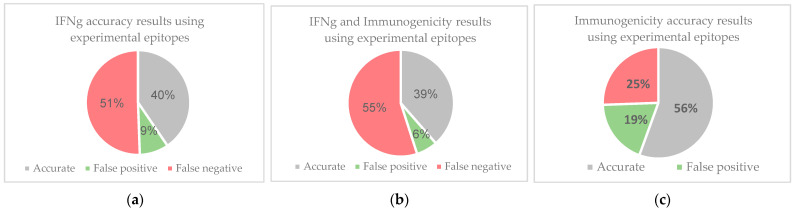
IFNepitope, Immunogenicity, and combined accuracy using experimental immunogenicity cancer epitopes with positive IFNγ release. Experimental data were collected from IEDB. (**a**) compares the predictions of IFNepitope to the results of experimental data. The data shows that 51% of IFNepitope’s data were found to be false negaetives, while 40% of epitopes were predicted accurately. (**b**) demonstrates that when considering the IFNγ release predictions of IFNepitope and the immunogenicity predictions of IEDB Immunogenicity, 55% of predictions are false negatives while 39% of the predictions are accurate. Most of these false negatives are the result of IFNepitope’s predictions. This can be seen in (**c**), which demonstrates that 56% of immunogenicity predictions using the IEDB immunogenicity tool are accurate, while 25% are false negatives and 19% are false positives. The data demonstrate that IFNepitope’s predictions for MHC class I peptides results in a high number of false negatives. As a result, the top epitopes were also determined without filtering for IFNγ release.

During the filtration process, many epitopes were eliminated due to the IFNγ filter, as they were predicted to be negative for IFNγ release. Despite the tool’s inherent design for MHC class II epitopes, accuracy tests using experimental epitopes were conducted on IFNepitope’s predictions on MHC class I molecules. Epitopes were retrieved from the Immune Epitope Database (IEDB) by filtering for linear peptides associated with cancer. These epitopes have positive T-cell assays for IFNγ release and result in positive cytokine activity. Using these parameters, querying the IEDB database resulted in 589 epitopes which can be found in Supplementary Table S4. Removing the parameters for cytokine activity and positive IFNγ release, resulted in 833 epitopes. IFNγ and immunogenicity predictions were made for the 833 epitopes (Supplementary Table S4). Epitopes from the 833-large group that were predicted to have positive IFNγ release and were also found in the 589-large group were considered accurate (Figure 7). Similarly, epitopes predicted to be negative for IFNγ release that were not found in the 589-large group were also considered accurate (Supplementary Table S4). The same accuracy determination methods were conducted on the data for immunogenicity and a combination of IFNγ release and immunogenicity.

### 3.11. Murine MHC Binding Affinity

Prior to human clinical trials, murine preclinical studies should be performed to measure the immune response to the vaccine in murine models. Therefore, we used a tool called NetH2pan to predict the binding affinity of the top epitopes to murine MHC molecules. The NetH2pan prediction tool was designed using over 8500 peptides eluted from FVB mice, and returns the binding affinity of the peptide to the MHC allele, as well as a qualitative prediction of strong binders and weak binders. Using this prediction tool, two epitopes were predicted to be weak binders to murine MHC molecules: LVVGADSV as a weak binder to H-2-Db and AGLGEYSAM as a weak binder to H-2-Dd and H-2-Kb. LVVVGADRV is predicted to be a strong binder to H-2-Db. Complete data for murine MHC binding prediction can be found in Supplementary Table S5.

### 3.12. Three-Dimensional (3D) Structural Analysis

**Figure 8:**
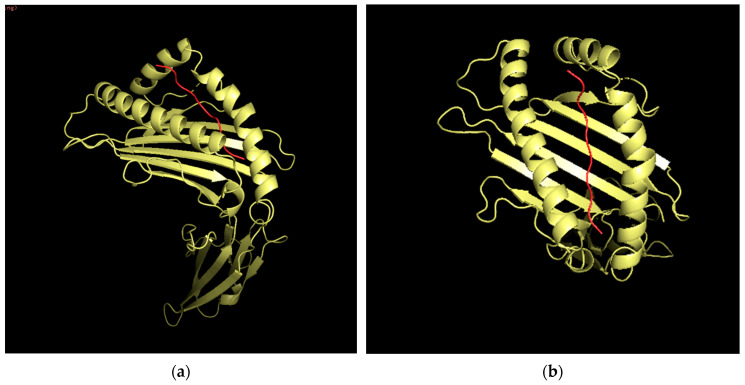
Three-dimensional structure of epitope VVVGACDVGK docked in HLA allele HLA-A*68:01. Red is the epitope sequence. Yellow is the HLA-A*68:01 molecule. (**a**) provides a frontal view of the MHC-peptide complex. (**b**) provides a top-down view of the MHC-peptide complex.

**Figure 9:**
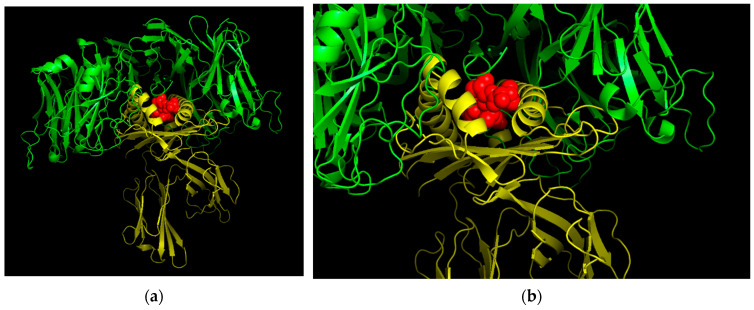
Three-dimensional structure of T36-5 TCR specific for HLA-A68. The TCR is modelled in green, the HLA allele is modelled in yellow, and the peptide is modelled in red. The HLA-peptide complex has been superimposed onto the TCR. (**a**) provides a full view of the TCR-MHC complex. (**b**) provides a zoomed in view of the complex that focuses on the MHC-peptide complex superimposed onto the TCR.

Three-dimensional analysis of the epitope VVVGACDVGK is displayed below, demonstrating the potential structure of the epitope when docking to an HLA allele (HLA-A*68:01 in this case). Information on the HLA allele structure was obtained from the RCSB protein data bank, while the structure of the epitope was determined using PEPstrMOD. The docking was conducted using PyMOL and the FlexPepDoc docking tool (Figure 8). An image of the HLA-peptide complex was superimposed onto a TCR for the HLA-A 68 allele (Figure 9).

## 4. Discussion

**Figure 10:**
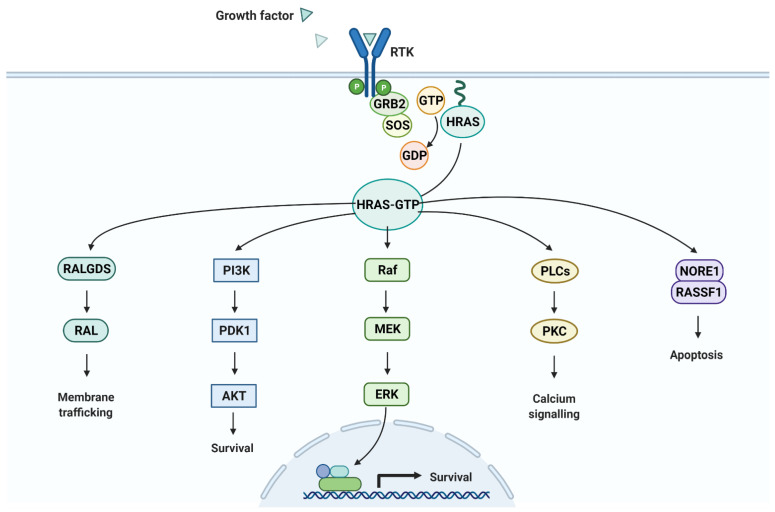
HRAS Pathways. The HRAS survival pathway including P13K and PDK1 has been extensively researched for its role in the proliferation of squamous cell carcinoma.

Many studies have been conducted on the HRAS, P13K, PDK1 pathway (Figure 10 [34]) [5,6,7]. These studies showed that either mutations or overexpression of these proteins can result in various cancers [5,6,7]. Sasaki et. al found that epitope vaccines designed for HRAS mutations did not decrease tumor size because patients were already in late stages of metastatic squamous cell carcinoma [5]. These vaccines only accounted for one mutation of HRAS, thereby limiting its flexibility. However, they did result in an immunogenic response, indicating the potential of HRAS as a target for therapeutic vaccines. Our study analyzes the top 15 HRAS mutations to create a vaccine, and analyzes several other clinically-relevant variables to increase the likelihood of inducing a reduction in tumor growth.

We developed a new epitope selection method which utilizes the clinically relevant-variables in vaccine design and applied this method to design HRAS epitope-based vaccine candidates. Using experimental epitopes, we determined that placing individual filters on each parameter was a superior method to creating a single quantitative measure to filter with. Furthermore, in silico prediction tools for several parameters (IFNγ release, allergenicity, immunogenicity) were compared to determine the optimal prediction tools for CD4 and CD8 epitopes. The tool ‘PCOptim’ is crucial in determining the success of the new filtration method, as it verified that placing individual filters on each parameter did not eliminate too many epitopes. Additionally, the PCOptim tool would provide guidance for future in silico peptide/epitope-based vaccine design, as it provides a dataset of comparison for any post-filtration epitope data.

Using the finalized filtration method, top mutated HRAS epitopes were selected. The final filtered epitopes, totaling 16 unique peptide sequences which potentially protect against nine unique HRAS mutations, are predicted to be immunogenic, antigenic, IFNγ releasing, non-toxic, non-allergenic, and stable. As a result of high allergenicity rates in epitopes with single mutations, many of the top epitopes include two simultaneous mutations. The predicted population coverage for these collective epitopes was adequately high, at 89.24% for the world population. However, the predicted population coverage across different regions of the world differ greatly with the highest being for Europe and North America. Clinically relevant epitope data for CD4 epitopes were collected and filtered following the same procedures used for the CD8 epitopes. However, due to the difficulty of obtaining high prediction accuracy for longer peptides, immunogenicity predictions could not be obtained and conclusions could not be drawn. The unfiltered data is available in Supplementary Table S6 and the filtered data can be found in Supplementary Table S7. The filtration can be validated using prediction tools that are more accurate for CD4 epitopes. 

Epitopes with single point mutations of G12D, G13C, and E62G do not provide any high-quality epitopes post-filtration. Several epitopes with these mutations are either predicted to be allergenic or do not release IFNγ. Combining mutations that occur on adjacent or nearby codons can provide epitopes that fit the outlined requirements to output a high-quality epitope. The prevalence of simultaneous mutations of the HRAS gene causing SCC is unknown, so further studies should be conducted on SCC patients with mutated HRAS gene.

The low post-filtration epitope recovery can be attributed to gene-specific attributes of Ras peptides. Many of the epitopes were eliminated during the filtration process due to the IFNγ predictions resulting in many negative predictions. To remedy this, another dataset selecting top epitopes while disregarding the IFNγ parameter was created following the results of the IFNepitope accuracy assessment using CD8 epitopes. This dataset can be accessed in Supplementary Table S8. It includes 64 unique peptides, although only six peptides are required to return maximum population coverage as determined by ‘PCOptim’. Total population coverage for this set of peptides is 98.55% as seen in Table 3. Additionally, regional population coverage is significantly higher than the more restricted dataset which includes IFNγ as a parameter. However, the coverage for central America remains low (Table 3). The IFNγ dataset may be a better fit to continue with for preclinical murine trials due to the limitations of the IFNepitope prediction tool. Another parameter of interest is the half-life of the peptide. The half-life of each peptide was determined using the ExPasy ProtParam tool, based on the N-terminal codon following the N-end rule. The data in this study report the half-life in vitro for mammalian reticulocytes, which is a limitation of the peptide selection method. Other methods to determine peptide half-life in vivo involve machine learning algorithms which analyze the individual amino acids within the peptide, as well as the dipeptide strings forming the overall peptide [35,36]. We did not use these prediction tools for this study as they are designed for longer peptide chains, and resulted in extremely short half-life predictions for the shorter peptides used in this study.

**Figure 11:**
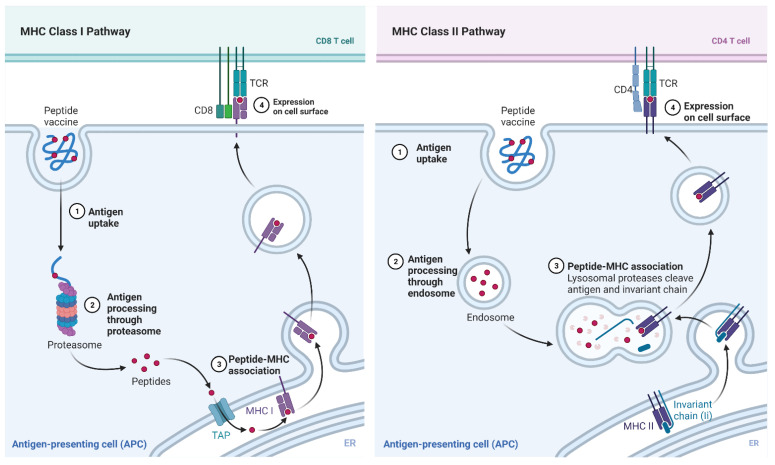
Peptide vaccine mechanism. Peptides will enter the cell and be presented superficially by MHC class I or II molecules. A CD8 T cell response will create an immediate immune response and a CD4 T-cell response will activate lymphocytes as well as create antigens for long-term immunity.

Due to the high immunogenicity and antigenicity scores of the individual peptides, the epitope-based vaccine is predicted to produce an immune response to both the initial immunization as well as the antigens post-vaccination. Figure 11 demonstrates the pathways taken by an epitope-based vaccine to elicit a T-cell response as well as produce antibodies for long-term immunity [37]. The combination of a CD8 and CD4 T-cell response will contribute to a broader, stronger and more long-lasting immune response. An uncertainty in the process of developing immunity is the peptide-MHC’s binding affinity to the T-cell receptor (TCR). The binding site of the TCR is extremely variable, so there are no binding prediction tools available for affinity between TCR, and an HLA molecule bound peptide. This issue is already slightly mitigated in this study, as the peptides have a predicted immunogenic response. However, the flaw can be further mitigated in murine preclinical studies by assessing whether the peptides result in an adequate immune response.

Previous studies involving the HRAS gene in designing epitope-based vaccines to target cancer have resulted in T-cell proliferation, but have failed to result in significant tumor size reduction [6]. The peptides selected through the filtration process designed in this study exhibit clinically relevant factors which serve as a positive indication of the clinical effects of this epitope-based vaccine. Furthermore, the epitopes selected in this study match the mutations identified in previous HRAS studies [5]. The same mutated G12C epitope which we identified in this study (Table 2) is also found with another previous study which identified the therapeutic abilities of HRAS-based peptide vaccines [3]. The G12C mutation is a common mutation in the Ras proto-oncogenes (HRAS, KRAS, and NRAS) and, therefore, might be a suitable mutation to target various types of cancer and their causes. This overlap confirms our epitope filtration method’s ability to select high-quality epitopes. The total number of epitopes identified in this study amounts to 16, increasing the likelihood of clinical success of a peptide-based vaccine. These epitopes account for more mutations of the HRAS gene as well as more HLA alleles.

## 5. Conclusions

The purpose of this study was to develop and implement a new epitopes selection method to predict clinically-viable epitopes for vaccine design. Several clinical checkpoints, such as immunogenicity, antigenicity, toxicity, IFNγ release, allergenicity, half-life, and stability, were predicted. The accuracy of these tools was determined either through literature review, or testing with experimental epitopes. Of the two tools tested for allergenicity predictions, AllerTop v2.0 was determined to be superior over AllerCatPro due to its higher sensitivity. IFNγ predictions using IFNepitope were deemed inaccurate for MHC class I epitopes, which led to the determination of top epitopes without filtering for positive IFNγ release. Previous studies have used IFNγ analysis in conjunction with docking analysis to select viable SARS-CoV-2 epitopes [27]. This epitope selection method can be broadly applied to other mutations for cancer-related genes to design epitope-based vaccines. Our optimization program PCOptim can be used in the design of other filtration methods, as it will provide a baseline necessity for top epitopes filtered. This can be useful in the development of epitope-based vaccines for cancer and non-cancer targets as well as other target proteins for squamous cell carcinoma. 

Using a dataset of SARS-CoV-2 immunogenic epitopes, we determined that the most effective method to select top epitopes was by placing filters on individual parameters. We verified this by filtering a dataset of SARS-CoV-2 non-immunogenic epitopes and comparing the top epitopes to a dataset of immunogenic SARS-CoV-2 epitopes. This method was implemented in the design of an epitope-based vaccine for HRAS mutations, resulting in 16 unique epitopes that account for nine unique mutations. Epitopes predicted to release IFNγ provide a world population coverage of 89.24%. Without considering epitope data for IFNγ release, 64 epitopes were selected resulting in 98.55% world population coverage. Additionally, the epitopes we selected using our filtration method were confirmed by matching them against the epitopes found in previous studies involving HRAS-based epitope vaccines.

A challenge in the design of an epitope-based vaccine exists during the stage of murine model development, as only one peptide was predicted to be a strong binder for murine MHC molecules, and two peptides were predicted as weak binders to three H2 alleles. While this study does predict clinically viable epitopes in humans, testing these epitopes using murine models may prove to be a challenge. One potential explanation for these results is that HRAS is not a suitable gene to perform murine model testing on, or that other HRAS mutations would result in better outcomes. Other potential mutations to investigate would include p53 and MUC1. These findings suggest that the newly designed filtration method is successful in accounting for clinically-relevant parameters to design an effective multi-valent epitope-based vaccine.

## Figures and Tables

**Table 1:** Parameters collected for each predicted epitope.

Parameter	Tool Name	Tool Link	Threshold
Rank	Immune Epitope Database (IEDB) NetMHCpan EL 4.1	http://tools.iedb.org/mhci/ (accessed on 24 September 2021)	<10
Immunogenicity	IEDB Immunogenicity	http://tools.iedb.org/immunogenicity/ (accessed on 24 September 2021)	>0
Antigenicity	VaxiJen	http://www.ddg-pharmfac.net/vaxijen/VaxiJen/VaxiJen.html (accessed on 24 September 2021)	>0.4
Half-Life	ProtParam	https://web.expasy.org/protparam/ (accessed on 24 September 2021)	>1 h
Toxicity	ToxinPred	https://webs.iiitd.edu.in/raghava/toxinpred/ (accessed on 24 September 2021)	Non-Toxic
IFNγ	IFNepitope	https://webs.iiitd.edu.in/raghava/ifnepitope/predict.php (accessed on 24 September 2021)	Positive
Allergenicity	Allertop v2.0	https://www.ddg-pharmfac.net/AllerTOP/ (accessed on 24 September 2021)	Non-Allergen
Isoelectric Point	ProtParam	https://web.expasy.org/protparam/ (accessed on 24 September 2021)	N/A
Instability Index	ProtParam	https://web.expasy.org/protparam/ (accessed on 24 September 2021)	<40
Aliphatic Index	ProtParam	https://web.expasy.org/protparam/ (accessed on 24 September 2021)	N/A
GRAVY Score	ProtParam	https://web.expasy.org/protparam/ (accessed on 24 September 2021)	N/A

**Table 2:** Summary of mutations and epitope sequences post-filtration.

Mutation	Peptide	HLA Alleles
G12C + G13D	VVVGACDVGK	HLA-A*68:01,HLA-A*11:01,HLA-A*03:01,HLA-A*30:01,HLA-A*31:01
G12D + G13C	KLVVVGADC	HLA-A*02:01
	LVVVGADCV	HLA-A*68:02,HLA-A*02:06,HLA-A*02:03,HLA-A*02:01
	VVVGADCVGK	HLA-A*68:01,HLA-A*11:01,HLA-A*03:01,HLA-A*30:01,HLA-A*31:01
G12D + G13D	KLVVVGADDV	HLA-A*02:03,HLA-A*02:01
	VVGADDVGK	HLA-A*11:01,HLA-A*68:01,HLA-A*03:01,HLA-A*30:01
	VVVGADDVGK	HLA-A*68:01,HLA-A*11:01,HLA-A*03:01
G12D + G13R	KLVVVGADR	HLA-A*31:01,HLA-A*03:01,HLA-A*68:01,HLA-A*33:01,HLA-A*11:01
	LVVVGADRV	HLA-A*68:02,HLA-A*02:06,HLA-A*02:03,HLA-A*02:01,HLA-B*51:01
G12D + G13S	KLVVVGADSV	HLA-A*02:03,HLA-A*02:01,HLA-A*02:06
	LVVVGADSV	HLA-A*02:06,HLA-A*68:02,HLA-A*02:03,HLA-B*51:01,HLA-A*02:01,HLA-A*26:01,HLA-B*35:01
G12S + G13C	KLVVVGASC	HLA-A*02:06,HLA-A*02:03,HLA-A*02:01,HLA-B*15:01,HLA-A*32:01
G13D	VVVGAGDVGK	HLA-A*11:01,HLA-A*68:01,HLA-A*03:01,HLA-A*30:01,HLA-A*31:01
Q61L	DTAGLEEYSA	HLA-A*68:02,HLA-A*26:01
Q61L + E62G	AGLGEYSAM	HLA-B*15:01,HLA-A*30:02,HLA-B*35:01,HLA-B*08:01,HLA-A*02:06,HLA-B*07:02,HLA-B*51:01,HLA-A*26:01
	DTAGLGEYSA	HLA-A*68:02,HLA-A*68:01,HLA-A*26:01

**Table 3:** Population coverage for regions of the world.

Area	Percent Coverage with IFNγ Filter	Percent Coverage without IFNγ Filter
Central Africa	68.24	86.04
Central America	2.78	7.76
East Africa	74.1	90.78
East Asia	85.33	98.18
Europe	94.32	99.68
North Africa	82.04	96.03
North America	90.7	99.06
Northeast Asia	83.73	94.7
Oceania	63.69	94.71
South Africa	75.77	93.03
South America	71.3	88.3
South Asia	83.44	94.73
Southeast Asia	72.0	94.56
Southwest Asia	80.94	92.5
West Africa	81.05	95.49
West Indies	88.11	98.98
World	89.24	98.55
Region Average	74.85	89.41
Standard Deviation	20.28	25.21

## Data Availability

Data supporting reported results and software code can be found in the Supplementary Materials.

## Supplementary Materials

The following are available online at https://www.mdpi.com/article/10.3390/vaccines10010063/s1. Supplementary Py Program 1. AllerTop v2.0 Webscraping Automation Program. Given an input of peptide sequences, the program will automatically retrieve data from the AllerTop v2.0 website and display the results to paste in a spreadsheet. Supplementary Py Program 2. ProtParam Webscraping Automation Program. Given an input of peptide sequences, the program will automatically retrieve data from the ProtParam website and display the results to paste in a spreadsheet. Supplementary Java Prog 1. PCOptim GUI Program. Given an input of epitopes and HLA allele data, the program will output the lowest number of alleles that are necessary to obtain the highest possible population coverage. Supplementary Java Prog 2. Mutation automation programs (all non-graphical user interface). Several automation programs are useful in obtaining full mutated peptide sequences, processing the summary output from the VaxiJen antigenicity tool, and printing inputted epitopes in FASTA format. Supplementary Table S1. Unfiltered HRAS epitope data. Supplementary Table S2. Immunogenic COVID epitopes raw data set and formula testing data. Supplementary Table S3. Top filtered HRAS epitopes with an IFNγ filter. Supplementary Table S4. IFNγ and Immunogenicity accuracy testing raw and processed data. Supplementary Table S5. Murine binding affinity data and predicted weak binders. Supplementary Table S6. Unfiltered HRAS MHCII epitope data. Supplementary Table S7. Filtered HRAS MHCII epitope data. Supplementary Table S8. Top filtered HRAS epitopes without an IFNγ filter.
