# Supplementary material for: A New Epitope Selection Method: Application to Design a Multi-Valent Epitope Vaccine Targeting HRAS Oncogene in Squamous Cell Carcinoma
Source: Vaccines (Basel). 2021 Dec 31;10(1):63. doi: 10.3390/vaccines10010063 (PMC8778118; doi:10.3390/vaccines10010063)
Supplement: Supplementary file 1 [file vaccines-10-00063-s001.zip › Supplemental Java Prog 2/FILENAME.html]

ILDTTGQEEY  
ILDTAGHEEY  
ILDTAGLEEY  
ILDTAGQGEY  
GEYSAMRDQY  
EYKLVVVGAR  
ILDTAGKEEY  
ILDTAGREEY  
VVGAGIVGK  
VVGAGVVGK  
VVGAVGVGK  
VVGDGGVGK  
RVGKSALTI  
VPMVLVGNKY  
VVGAGSVGK  
VVGASGVGK  
VVGAAGVGK  
VVGARGVGK  
VVVGAGIVGK  
VVGAGRVGK  
VVVGDGGVGK  
KYDLAARTV  
VVVGAGVVGK  
GARGVGKSAL  
AGRVGKSAL  
VVVGAGSVGK  
VVVGASGVGK  
KLVVVGAGV  
NLAARTVESR  
DVGKSALTI  
KLVVVGAGR  
SVGKSALTI  
VVVGARGVGK  
VVVGAGRVGK  
VVVGAAGVGK  
AAGVGKSAL  
VVGACGVGK  
LVVVGAVGV  
AVGVGKSAL  
VVGAGCVGK  
VVGKSALTI  
VVVGACGVGK  
VVVGADGVGK  
VVVGAGCVGK  
VVVGAGDVGK  
VVGAGDVGK  
IVGKSALTI  
VVVGAVGVGK  
LDTTGQEEY  
AGKEEYSAM  
DTAGQGEYSA  
LVVVGAAGV  
DTAGLEEYSA  
LDTAGQGEY  
GAGRVGKSAL  
DTAGHEEYSA  
LDTAGHEEY  
AGREEYSAM  
LDTAGLEEY  
CLLDILDTT  
PMVLVGNKY  
DGVGKSALTI  
LVVVGASGV  
AGDVGKSAL  
AGSVGKSAL  
DTAGKEEYSA  
DGGVGKSAL  
KLVVVGAGI  
LDTAGKEEY  
LDTAGREEY  
TGQEEYSAM  
GNKYDLAAR  
AGIVGKSAL  
TAGHEEYSAM  
AGKEEYSAMR  
GARGVGKSA  
KLVVVGASGV  
DTAGREEYSA  
VLVGNKYDL  
GAAGVGKSAL  
ASGVGKSAL  
RVGKSALTIQ  
AGVVGKSAL  
TAGLEEYSAM  
TTGQEEYSAM  
GQGEYSAMR  
LVVVGADGV  
KLVVVGAVGV  
AGREEYSAMR  
GNKCNLAAR  
KLVVVGAAGV  
KLVVVGAGSV  
LVVVGARGV  
GASGVGKSAL  
LLDILDTAGL  
VLVGNKCNL  
KLVVVGARGV  
VGNKYDLAAR  
AGHEEYSAM  
DTAGLEEYS  
AGQGEYSAMR  
GADGVGKSAL  
TAGREEYSAM  
GEYSAMRDQ  
RGVGKSALTI  
KLVVVGAGRV  
TAGQGEYSAM  
ADGVGKSAL  
DTAGHEEYS  
DTAGKEEYS  
TAGKEEYSAM  
LDILDTAGL  
TCLLDILDTT  
DTAGQGEYS  
KLVVVGACGV  
KLVVVGADGV  
GAVGVGKSAL  
LLDILDTAGK  
VGNKCNLAAR  
KLVVVGAGCV  
LVVVGAGSV  
YKLVVVGAGV  
KLVVVGAGVV  
AGHEEYSAMR  
KLVVVGAGDV  
GAGDVGKSAL  
KLVVVGAGIV  
NLAARTVES  
LVVVGACGV  
CVGKSALTI  
GIVGKSALTI  
GAGSVGKSAL  
TTGQEEYSA  
GVVGKSALTI  
AGLEEYSAMR  
DTAGREEYS  
LVVVGAGRV  
ILDTAGLEE  
ACGVGKSAL  
GLEEYSAMR  
LLDILDTAGH  
ILDTTGQEE  
DTTGQEEYSA  
AGCVGKSAL  
GAGIVGKSAL  
GSVGKSALTI  
KLVVVGDGGV  
GDGGVGKSAL  
NKYDLAARTV  
VLVGNKCNLA  
ARGVGKSAL  
YKLVVVGAGR  
TGQEEYSAMR  
GIVGKSALT  
AGQGEYSAM  
AGLEEYSAM  
LDILDTAGR  
YKLVVVGAR  
YDLAARTVES  
GAGVVGKSAL  
VLVGNKYDLA  
GVVGKSALT  
ILDTAGHEE  
GACGVGKSAL  
GDVGKSALTI  
VVGAGIVGKS  
GHEEYSAMR  
LVVVGAGVV  
VVGDGGVGKS  
LVVVGAGCV  
KCNLAARTV  
GAAGVGKSA  
VVGAGVVGKS  
YKLVVVGAGI  
VVGAVGVGKS  
LDILDTAGK  
LLDILDTAGR  
GREEYSAMR  
LVVVGDGGV  
VVGASGVGKS  
LVVVGAGIV  
VGARGVGKSA  
GASGVGKSA  
ILDTAGQGE  
QGEYSAMRDQ  
VVGAAGVGKS  
GAGCVGKSAL  
VVGAGSVGKS  
MVLVGNKCNL  
VVGAGRVGKS  
SVGKSALTIQ  
GAVGVGKSA  
MVLVGNKYDL  
TAGQGEYSA  
TAGHEEYSA  
DTTGQEEYS  
EYKLVVVGAA  
VVGARGVGKS  
DGVGKSALT  
GADGVGKSA  
HEEYSAMRDQ  
YKLVVVGAV  
KLVVVGAGC  
VGVGKSALTI  
EYKLVVVGAV  
ILDTAGKEE  
LVGNKCNLA  
TAGKEEYSA  
GKEEYSAMR  
LVVVGAGDV  
RGVGKSALT  
KLVVVGAGS  
SGVGKSALTI  
TAGLEEYSA  
GAGSVGKSA  
REEYSAMRDQ  
LLDILDTTGQ  
AGIVGKSALT  
VVGADGVGKS  
DVGKSALTIQ  
VGAAGVGKSA  
AVGVGKSALT  
AGVVGKSALT  
KEEYSAMRDQ  
TEYKLVVVGD  
GDGGVGKSA  
LVGNKYDLA  
EYKLVVVGAS  
KLVVVGAAG  
VVGKSALTIQ  
DILDTTGQE  
EYKLVVVGAC  
GACGVGKSA  
KLVVVGAVG  
ILDTAGREE  
VGADGVGKSA  
VVGAGDVGKS  
IVGKSALTIQ  
GAGRVGKSA  
TAGREEYSA  
KYDLAARTVE  
VGNKCNLAA  
VGAVGVGKSA  
GAGDVGKSA  
GDVGKSALT  
GAGIVGKSA  
GSVGKSALT  
LLDILDTTG  
LVGNKYDLAA  
CNLAARTVES  
CGVGKSALTI  
SGVGKSALT  
HEEYSAMRD  
LDTTGQEEYS  
DILDTAGQG  
YKLVVVGAA  
AGVGKSALT  
VVGACGVGKS  
VGASGVGKSA  
GCVGKSALTI  
AGRVGKSALT  
GAGVVGKSA  
DILDTAGHE  
DILDTAGHEE  
DILDTAGLE  
REEYSAMRD  
AGVGKSALTI  
YKLVVVGAC  
DILDTAGLEE  
VGNKYDLAA  
KLVVVGASG  
VVVGAVGVG  
YKLVVVGAGC  
VVGAGCVGKS  
VGAGRVGKS  
YKLVVVGAGS  
VVVGAGSVG  
VGAGSVGKSA  
DILDTAGKE  
KEEYSAMRD  
LEEYSAMRDQ  
VGDGGVGKSA  
CLLDILDTTG  
LDTAGQGEYS  
AAGVGKSALT  
KLVVVGARG  
VGVGKSALT  
VGAGIVGKS  
VGAGIVGKSA  
VVVGAGRVG  
VVVGAGVVG  
VGAGVVGKS  
LDTAGHEEYS  
DILDTAGRE  
DILDTTGQEE  
NKCNLAARTV  
VGACGVGKSA  
VGAGRVGKSA  
VGAGVVGKSA  
LDTAGREEYS  
VVVGASGVG  
VGASGVGKS  
VVVGAAGVG  
CVGKSALTIQ  
VVVGAGIVG  
GRVGKSALTI  
VGAGSVGKS  
LDTAGKEEYS  
LDTAGLEEYS  
DILDTAGREE  
VGAGDVGKSA  
GNKYDLAART  
KCNLAARTVE  
DILDTAGQGE  
VGAGCVGKSA  
VGAAGVGKS  
VGADGVGKS  
VVVGARGVG  
YKLVVVGASG  
GAGCVGKSA  
EYKLVVVGD  
MVLVGNKYD  
NKYDLAART  
GQGEYSAMRD  
KLVVVGACG  
VGARGVGKS  
GCVGKSALT  
LVGNKCNLAA  
YKLVVVGAAG  
EYKLVVVGAD  
LDILDTAGH  
VVVGDGGVG  
LVVVGASGVG  
ASGVGKSALT  
AGSVGKSALT  
GLEEYSAMRD  
LEEYSAMRD  
VGDGGVGKS  
LVVVGAAGVG  
VVVGACGVG  
YKLVVVGARG  
LVVVGAGSVG  
DGGVGKSALT  
GNKCNLAART  
CGVGKSALT  
VVVGAGCVG  
KLVVVGAGD  
AGDVGKSALT  
YKLVVVGAVG  
VGAVGVGKS  
VVVGAGDVG  
PMVLVGNKYD  
KLVVVGADG  
KLVVVGDGG  
VVVGADGVG  
ARGVGKSALT  
LVVVGAVGVG  
VGAGCVGKS  
VGAGDVGKS  
LDILDTTGQ  
LVVVGARGVG  
GRVGKSALT  
DILDTAGKEE  
LVVVGAGVVG  
CNLAARTVE  
LDILDTTGQE  
LVVVGACGVG  
LDILDTAGRE  
GHEEYSAMRD  
AGCVGKSALT  
LDILDTAGLE  
VGACGVGKS  
YKLVVVGAS  
YKLVVVGDGG  
ACGVGKSALT  
LVVVGADGVG  
GKEEYSAMRD  
YKLVVVGACG  
ADGVGKSALT  
MVLVGNKCN  
LDILDTAGHE  
LDILDTAGKE  
GREEYSAMRD  
YKLVVVGADG  
YKLVVVGAGD  
EYKLVVVGDG  
YDLAARTVE  
LVVVGAGIVG  
LVVVGAGRVG  
LDILDTAGQG  
QGEYSAMRD  
NKCNLAART  
LVVVGDGGVG  
LVVVGAGCVG  
LVVVGAGDVG  
YKLVVVGAD  
YKLVVVGDG  
PMVLVGNKCN  
